# HBFP: a new repository for human body fluid proteome

**DOI:** 10.1093/database/baab065

**Published:** 2021-10-11

**Authors:** Dan Shao, Lan Huang, Yan Wang, Xueteng Cui, Yufei Li, Yao Wang, Qin Ma, Wei Du, Juan Cui

**Affiliations:** Department of Computer Science and Engineering, University of Nebraska-Lincoln, 122E Avery Hall, 1144 T St., Lincoln, NE 68588, USA; Key Laboratory of Symbol Computation and Knowledge Engineering of Ministry of Education, College of Computer Science and Technology, Jilin University, 2699 Qianjin Street, Changchun 130012, China; Department of Computer Science and Technology, Changchun University, 6543 Weixing Road, Changchun 130022, China; Key Laboratory of Symbol Computation and Knowledge Engineering of Ministry of Education, College of Computer Science and Technology, Jilin University, 2699 Qianjin Street, Changchun 130012, China; Key Laboratory of Symbol Computation and Knowledge Engineering of Ministry of Education, College of Computer Science and Technology, Jilin University, 2699 Qianjin Street, Changchun 130012, China; Department of Computer Science and Technology, Changchun University, 6543 Weixing Road, Changchun 130022, China; Department of Computer Science and Technology, Changchun University, 6543 Weixing Road, Changchun 130022, China; Key Laboratory of Symbol Computation and Knowledge Engineering of Ministry of Education, College of Computer Science and Technology, Jilin University, 2699 Qianjin Street, Changchun 130012, China; Department of Biomedical Informatics, College of Medicine, The Ohio State University, 310G Lincoln tower, 1800 cannon drive, Columbus, OH 43210, USA; Key Laboratory of Symbol Computation and Knowledge Engineering of Ministry of Education, College of Computer Science and Technology, Jilin University, 2699 Qianjin Street, Changchun 130012, China; Department of Computer Science and Engineering, University of Nebraska-Lincoln, 122E Avery Hall, 1144 T St., Lincoln, NE 68588, USA

## Abstract

Body fluid proteome has been intensively studied as a primary source for disease biomarker discovery. Using advanced proteomics technologies, early research success has resulted in increasingly accumulated proteins detected in different body fluids, among which many are promising biomarkers. However, despite a handful of small-scale and specific data resources, current research is clearly lacking effort compiling published body fluid proteins into a centralized and sustainable repository that can provide users with systematic analytic tools. In this study, we developed a new database of human body fluid proteome (HBFP) that focuses on experimentally validated proteome in 17 types of human body fluids. The current database archives 11 827 unique proteins reported by 164 scientific publications, with a maximal false discovery rate of 0.01 on both the peptide and protein levels since 2001, and enables users to query, analyze and download protein entries with respect to each body fluid. Three unique features of this new system include the following: (i) the protein annotation page includes detailed abundance information based on relative qualitative measures of peptides reported in the original references, (ii) a new score is calculated on each reported protein to indicate the discovery confidence and (iii) HBFP catalogs 7354 proteins with at least two non-nested uniquely mapping peptides of nine amino acids according to the Human Proteome Project Data Interpretation Guidelines, while the remaining 4473 proteins have more than two unique peptides without given sequence information. As an important resource for human protein secretome, we anticipate that this new HBFP database can be a powerful tool that facilitates research in clinical proteomics and biomarker discovery.

**Database URL:** https://bmbl.bmi.osumc.edu/HBFP/

## Background

Human body fluids are thought to be rich resources of disease-associated proteins that are secreted or leaked from pathological tissues across the body, many of which are commonly obtainable through non-invasive procedures (1, 2). Driven by these factors, research interests have soared a few decades ago toward biomarker discovery by examining body fluid proteomes. It is highly plausible that empowered by innovative high-throughput technologies, modern proteomic studies have successfully identified a large number of proteins in various body fluids such as plasma, serum, saliva and urine (3).

With great effort by a few large consortiums, several community-based proteomic databases have been developed in the past decades. For example, in 2002, the international Human Proteome Organization initiated the Human Plasma Proteome Project and reported human plasma and serum protein constituents in its online databases (4). Another similar database, named Plasma Proteome Database, archived more than 10 000 proteins detected in human blood (5). Additionally, the Proteomics Identifications database (6) and Human Plasma PeptideAtlas (7) report a total of 3509 high-confidence plasma proteins. More recently, the extracellular vesicles community also reports new proteins identified in exosomes in multiple different resources including blood and breast milk, e.g. in ExoCarta (8). Additionally, the global Human Proteome Project (HPP) announces a set of mass spectrometry (MS) data interpretation guidelines that are presented to the broader research community (9).

Our team has recently conducted a systematical assessment of human proteome identified using quantitative proteomics tools such as MS and computational predictive models, as documented in a recent review article (10). To expand this effort, we developed a new human body fluid proteome (HBFP) database to organize 11 827 unique proteins reported in 164 scientific articles since 2001, which has a maximal false discovery rate (FDR) of 0.01 on both the peptide and protein levels. Until today, this database stores information about proteins from 17 types of body fluids including plasma/serum, saliva, urine, cerebrospinal fluid (CSF), seminal fluid (SF), amniotic fluid, tear fluid, bronchoalveolar lavage fluid (BALF), milk, synovial fluid, nipple aspirate fluid, cervical-vaginal fluid, pleural effusion, sputum, exhaled breath condensate, pancreatic juice and sweat. For each protein entry, description about protein secretion information, literature source, abundances, confidence and functional annotation is provided. This database system also provides users easy access to data visualization and download and functional analysis based on Gene Ontology (GO) and pathways.

## Database content and design

### Protein entries

We have manually collected proteins reported in 17 types of body fluids by carefully reviewing 164 scientific references published since 2001 based on a PubMed search with FDR ≤1% on both the peptide and protein levels.

**Figure 1.**
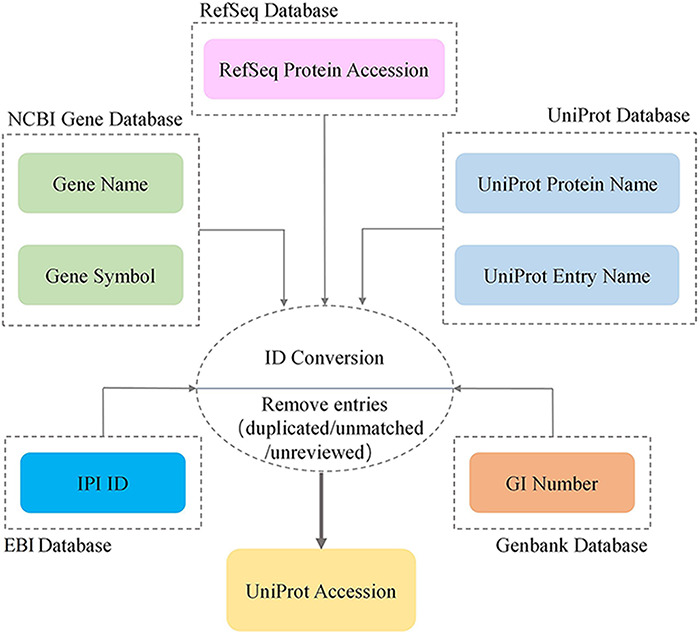
Workflow of protein identifier conversion.

In the HBFP database, each protein is assigned with a unique identifier of UniProtKB/Swiss-Prot accession (UniProt release 2020_06) (11). Since different identifiers have been mixed used in the referenced studies, we first used conversion tools at BioDBnet (https://biodbnet-abcc.ncifcrf.gov/) (12) and UniProt (https://www.UniProt.org/) to confidently convert different identifiers to UniProt accession numbers. The common identifiers involved in this study include International Protein Index ID [hosted at European Bioinformatics Institute (EBI) (closed in 2011)], GI number (from Genbank database), RefSeq protein accession (from RefSeq database), Gene name/symbol (from NCBI Gene database) and UniProt protein/entry name (from UniProt database). The ID conversion process is shown in Figure 1. During the conversion, poorly curated proteins with ambiguous identifiers were eliminated. For examples, many International Protein Index ID links to unclearly described instances that cannot be mapped to a UniProt entry are excluded.

### Database utilities

The interface of the HBFP database is constructed by PHP, while the database system is based on MySQL. The main contents of the current database include query and browse pages described as follows.

#### Querying page

**Figure 2.**
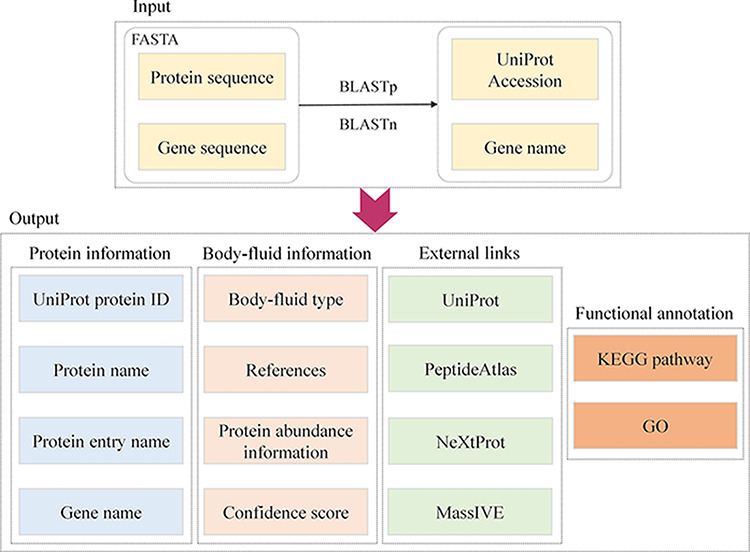
Construction workflow and utilities of querying page.

As one of the most important functions, the querying page allows users to search for body fluid proteins based on different types of input including protein ID, gene name, and protein or gene sequence. When given a FASTA input, BLASTp or BLASTn is used to translate sequence input to the best-match protein entry. The top hit (the highest bit score) from the BLAST search is considered the best match of the query. Figure 2 illustrates the workflow and content of querying page.

The annotation of each protein contains the following information:

- Protein ID/name/entry name
- Gene name
- Associated body fluid type along with indicated discovery confidence (explained in the next section)
- References and protein abundance information where the protein is reported
- External links to public databases including UniProt, PeptideAtlas and NeXtProt (13), MassIVE (14)
- Functional annotation based on the KEGG pathway (15) and GO (16)

#### Browsing page

This page provides an overview list of proteins associated with 17 types of body fluids and links to view and download selected proteins.

## Database highlights

### Data statistics

When determining the inclusion of reported proteins, we applied the following criteria for credibility of the MS evidence. First, for papers that issued peptide sequence details, we remapped all those peptide sequences to neXtProt (release 2021-02-15) using the neXtProt peptide uniqueness checker to remove unreliable matches (17). Specifically, we applied guideline #15 of HPP Guidelines 2.1 (9) to include proteins that contain at least two non-nested uniquely mapping peptides of nine amino acids into the HBFP database. According to this criterion, 7354 proteins were confirmed confidently. Another 4473 proteins were also included as they were not explicitly provided with peptide sequence information but have more than two unique peptides.

The overall statistics about the protein entries and references in terms of each body fluid are summarized in Table 1. The current HBFP database contains 11 827 distinct proteins from 17 types of body fluids. Note that urine exceeds all other body fluids in terms of protein counts while blood is at the second rank. All data are made publicly available in the HBFP and via links at https://bmbl.bmi.osumc.edu/HBFP/.

**Table 1.** Overall statistics

		Statistics		
Body fluid types		Number of protein entries	Number of references	References
1	Plasma/serum	5790	38	(18–55)
2	Saliva	2758	21	(19, 56–75)
3	Urine	7330	23	(19, 76–97)
4	CSF	4364	12	(19, 90, 98–107)
5	SF	4084	5	(108–112)
6	Amniotic fluid	3025	6	(19, 113–117)
7	Tear fluid (TF)	1882	11	(118–128)
8	BALF	3434	6	(41, 129–133)
9	Milk	2457	14	(134–147)
10	Synovial fluid	1637	7	(148–154)
11	Nipple aspirate fluid	1734	5	(155–159)
12	Cervical–vaginal fluid	949	4	(160–163)
13	Pleural effusion	1519	3	(164–166)
14	Sputum	1809	3	(167–169)
15	Exhaled breath condensate	351	5	(170–174)
16	Pancreatic juice	702	4	(175–178)
17	Sweat	1244	3	(179–181)
Total (non-redundant)		11 827	164	

### Protein abundance

**Figure 3.**
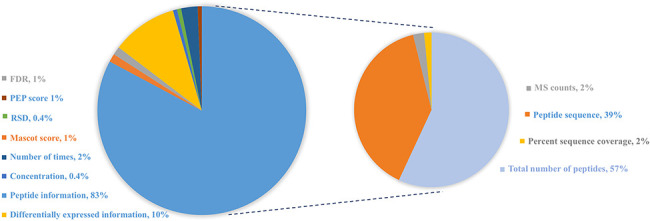
Distribution of protein abundance methods in HBFP database based on a number of original quantitative analysis methods from the original literature studies. Note that the sum of protein abundance is not 100% since not all of the literature studies provide quantitative analysis information.

In order to provide users experimental evidence from the original study, this database also displays relatively abundant information from the corresponding literature studies. General proteomics approaches using MS identify proteins by matching identified peptides against predefined protein sequence databases. The qualitative measures of protein reported in the original reference include the following: (i) peptide information: most of cited studies provide explicit information about peptide sequence, the total number of peptides, MS counts or the percent sequence coverage; (ii) differential expression information including fold change (positive value demonstrates up-regulated expression and negative value indicates down-regulated expression), up- or down-regulated expression in case vs. control or (normalized) spectral counts and (iii) other statistical information including FDR, relative standard deviation and the number of times across different samples or experiments, as shown in Figure 3.

### Confidence score

In the HBFP database, to evaluate the confidence level of each discovered protein in each body fluid, a new statistical measure is calculated based on Guideline # 9 of HPP guidelines 2.1 for the combined datasets. It is a well-known phenomenon that when taking *N* datasets with a substantial FDR and piling them all together, the overall FDR increases with the number of datasets. For example, for plasma, there are 38 papers with plasma protein lists, each with a substantial FDR ($ \le $1%). It is probably a conservative estimate to suppose that the FDR of such a combined result is 1% + 0.5%$ \times $(*N* datasets$ - $1) (9). It means that 50% of the correct identifications overlap and none of the incorrect ones does, so the resulting FDR is added in a 0.5% increment. Meanwhile, the confidence level of protein in the combined datasets is also reduced. Otherwise, considering the overlap of the true positives, the larger the number of datasets in which a protein is associated with a specific fluid, the more reliable this protein is. In the end, a confidence score $C$ is calculated as follows:

(1) \begin{equation*}{C_{i,j}} = {A_i} + 0.5\% \times \left( {{M_j} - 1} \right)\end{equation*}

(2) \begin{equation*}{A_i} = 1 - FD{R_i}\end{equation*}

(3) \begin{equation*}FD{R_i} = 1\% + 0.5\% \times \left( {{N_i} - 1} \right)\vspace*{6pt}\end{equation*}

where ${N_i}$ is the number of relevant literature studies (datasets) of a specific fluid $i$; $FD{R_i}$ represents the overall FDR of multiple datasets in body fluid $i$; ${A_i}$ means the confidence level of proteins in the combined datasets of body fluid $i$ and ${M_j}$ refers to the number of literature studies in which a protein $j$ is identified in body fluid $i$.

For example, there are 38 literature studies related to blood in the HBFP, so ${N_i} = 38$, $FD{R_i} = 0.195$ and ${A_i} = 0.805$. The protein O14791 is identified in blood by 19 independent studies, i.e. ${M_j} = 19$. As a result, the calculated ${C_{i,j}}$ score for O14791 in blood is $0.895$. Meanwhile, protein Q9UJV9 only is identified in one paper for blood, so ${M_j} = 1$ and ${C_{i,j}} = {A_i} = 0.805$. It means that protein Q9UJV9 maintains only the confidence level in the combined datasets of blood. Specifically, protein P01833 is identified in milk by 14 studies, and a total of 14 literature studies on milk are included in the HBFP, so protein P01833 maintains the original confidence level, i.e. 0.99. The larger the $C$ score, the higher the confidence that a protein reported in that fluid will be. Note that this score can only be compared within the same type of body fluid.

## Database applications

### Data access

The website can be accessed through https://bmbl.bmi.osumc.edu/HBFP/.

#### Query

**Figure 4.**
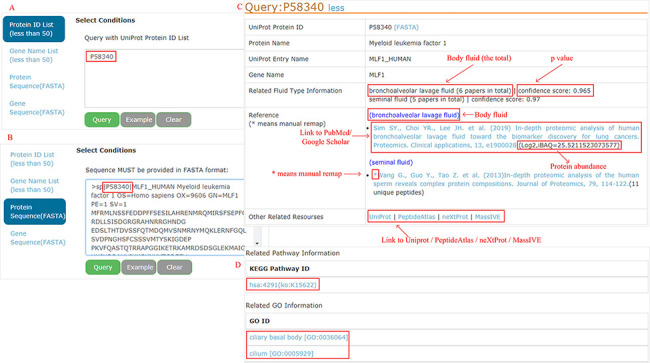
Example of query response with input as ‘P58340’ in the protein ID and protein sequence box.

All proteins can be easily accessed by searching protein ID, gene name, protein sequence (FASTA) or gene sequence (FASTA) (<50 items per query) (Figure 4A and B as an example). A BLAST (182) is performed locally to find the best match when the sequence FASTA format is given. For each protein, detailed information is displayed (Figure 4C). Users can connect directly to the PubMed or Google Scholar to view the original study through the provided links. Four databases (UniProt, PeptideAtlas, NeXtProt and MassIVE) are cross-linked for additional protein annotation, while the KEGG pathway and GO are focused on the functional aspects (Figure 4D).

#### Download

**Figure 5.**
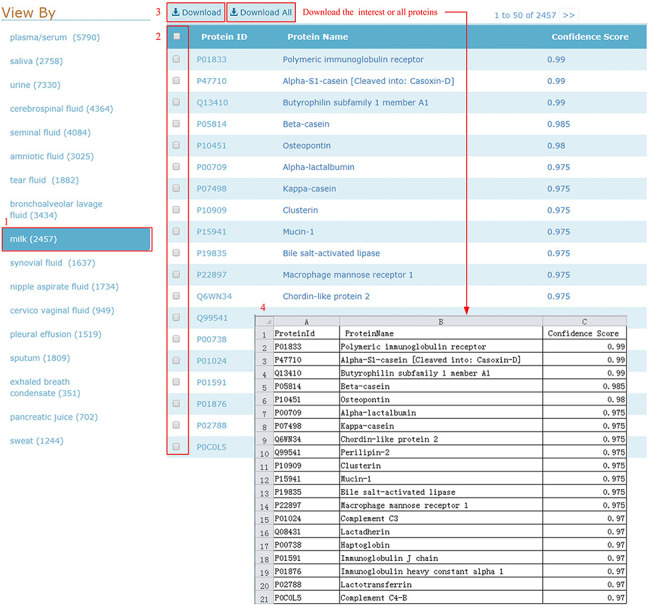
Download illustration where user can choose the body fluid name and download the proteins of interest or all proteins.

HBFP allows users to browse the entire protein list in each body fluid, where the proteins are ordered based on descending confidence scores. Users can check and download all entries of the selected body fluid type in one go, as shown in Figure 5.

### Demo of comparative analysis using the HBFP database

#### Body fluid analysis

**Figure 6.**
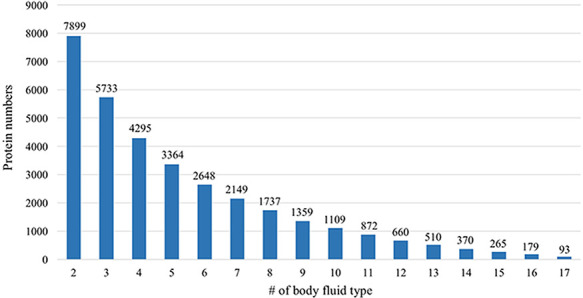
Comparative analysis across different body fluids. Seven thousand eight hundred and ninety-nine (7899) proteins are presented in at least two body fluids and 5733 proteins existed in at least three body fluids. Only 93 proteins exist in all 17 body fluids.

Many proteins in the HBFP database have a broad distribution in terms of body fluid types. An internal comparative analysis across different fluids can provide further information regarding the specificity of a proposed marker protein. Of 11 827 identified proteins, 66.8% are identified in at least two body fluids (Figure 6). A total of 93 proteins (0.79%) are shared among all analyzed body fluids, which may indicate that these proteins are essential for various life activities (Table 2).

**Table 2.** List of 93 proteins shared among all 17 body fluids

	UniProt accession number	Protein name	Gene name
1	P11021	Endoplasmic reticulum chaperone BiP	HSPA5
2	P55072	Transitional endoplasmic reticulum ATPase	VCP
3	P13647	Keratin, type II cytoskeletal 5	KRT5
4	O00299	Chloride intracellular channel protein 1	CLIC1
5	P02787	Serotransferrin	TF
6	P22314	Ubiquitin-like modifier-activating enzyme 1	UBA1
7	P13645	Keratin, type I cytoskeletal 10	KRT10
8	P02533	Keratin, type I cytoskeletal 14	KRT14
9	P07237	Protein disulfide-isomerase	P4HB
10	P06576	ATP synthase subunit beta, mitochondrial	ATP5F1B
11	P30041	Peroxiredoxin-6	PRDX6
12	P63104	14-3-3 protein zeta/delta	YWHAZ
13	P62258	14-3-3 protein epsilon	YWHAE
14	P14923	Junction plakoglobin	JUP
15	P04040	Catalase	CAT
16	P01834	Immunoglobulin kappa constant	IGKC
17	P06702	Protein S100-A9	S100A9
18	P52209	6-Phosphogluconate dehydrogenase, decarboxylating	PGD
19	P18669	Phosphoglycerate mutase 1	PGAM1
20	P14618	Pyruvate kinase PKM	PKM
21	P61981	14-3-3 protein gamma	YWHAG
22	P07384	Calpain-1 catalytic subunit	CAPN1
23	P50395	Rab GDP dissociation inhibitor beta	GDI2
24	Q00610	Clathrin heavy chain 1	CLTC
25	P26641	Elongation factor 1-gamma	EEF1G
26	P32119	Peroxiredoxin-2	PRDX2
27	P19971	Thymidine phosphorylase	TYMP
28	P26038	Moesin	MSN
29	P40121	Macrophage-capping protein	CAPG
30	P35754	Glutaredoxin-1	GLRX
31	P01009	Alpha-1-antitrypsin	SERPINA1
32	P01860	Immunoglobulin heavy constant gamma 3	IGHG3
33	P06753	Tropomyosin alpha-3 chain	TPM3
34	P68871	Hemoglobin subunit beta	HBB
35	P62805	Histone H4	H4C1
36	P30086	Phosphatidylethanolamine-binding protein 1	PEBP1
37	P35579	Myosin-9	MYH9
38	P01023	Alpha-2-macroglobulin	A2M
39	Q06830	Peroxiredoxin-1	PRDX1
40	P02042	Hemoglobin subunit delta	HBD
41	P07737	Profilin-1	PFN1
42	P80188	Neutrophil gelatinase-associated lipocalin	LCN2
43	P02679	Fibrinogen gamma chain	FGG
44	P40925	Malate dehydrogenase, cytoplasmic	MDH1
45	P08758	Annexin A5	ANXA5
46	P46940	Ras GTPase-activating-like protein IQGAP1	IQGAP1
47	P01833	Polymeric immunoglobulin receptor	PIGR
48	P31949	Protein S100-A11	S100A11
49	P04792	Heat shock protein beta-1	HSPB1
50	P07339	Cathepsin D	CTSD
51	P01857	Immunoglobulin heavy constant gamma 1	IGHG1
52	P06733	Alpha-enolase	ENO1
53	P23284	Peptidyl-prolyl cis-trans isomerase B	PPIB
54	P02647	Apolipoprotein A-I	APOA1
55	O43707	Alpha-actinin-4	ACTN4
56	P30740	Leukocyte elastase inhibitor	SERPINB1
57	Q16610	Extracellular matrix protein 1	ECM1
58	P60709	Actin, cytoplasmic 1	ACTB
59	P15924	Desmoplakin	DSP
60	P62937	Peptidyl-prolyl cis-trans isomerase A	PPIA
61	P17931	Galectin-3	LGALS3
62	P00491	Purine nucleoside phosphorylase	PNP
63	P04080	Cystatin-B	CSTB
64	P02788	Lactotransferrin	LTF
65	P13639	Elongation factor 2	EEF2
66	P35527	Keratin, type I cytoskeletal 9	KRT9
67	P06396	Gelsolin	GSN
68	P59998	Actin-related protein 2/3 complex subunit 4	ARPC4
69	P25311	Zinc-alpha-2-glycoprotein	AZGP1
70	P02768	Albumin	ALB
71	P61160	Actin-related protein 2	ACTR2
72	P04406	Glyceraldehyde-3-phosphate dehydrogenase	GAPDH
73	P60174	Triosephosphate isomerase	TPI1
74	P18206	Vinculin	VCL
75	P08670	Vimentin	VIM
76	P10599	Thioredoxin	TXN
77	P11142	Heat shock cognate 71 kDa protein	HSPA8
78	P01011	Alpha-1-antichymotrypsin	SERPINA3
79	P04075	Fructose-bisphosphate aldolase A	ALDOA
80	P04264	Keratin, type II cytoskeletal 1	KRT1
81	P37837	Transaldolase	TALDO1
82	P35908	Keratin, type II cytoskeletal 2 epidermal	KRT2
83	P02545	Prelamin-A/C	LMNA
84	P69905	Hemoglobin subunit alpha	HBA1
85	P07900	Heat shock protein HSP 90-alpha	HSP90AA1
86	P29401	Transketolase	TKT
87	P00558	Phosphoglycerate kinase 1	PGK1
88	P00338	L-lactate dehydrogenase A chain	LDHA
89	P01861	Immunoglobulin heavy constant gamma 4	IGHG4
90	P05109	Protein S100-A8	S100A8
91	P04083	Annexin A1	ANXA1
92	P01024	Complement C3	C3
93	P09211	Glutathione S-transferase P	GSTP1

#### Venn diagram and GO annotation

**Figure 7.**
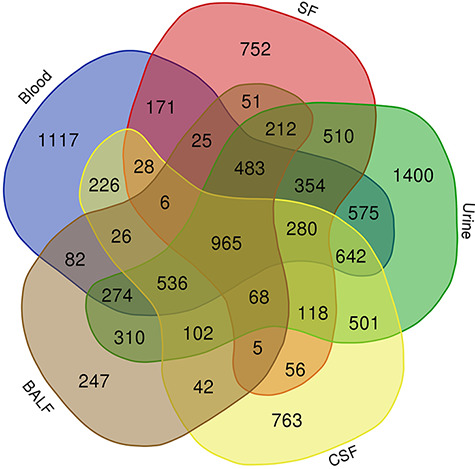
Venn diagram showing the common proteins among five body fluids (blood, urine, CSF, SF and BALF) that have the most number of proteins in the HBFP.

**Figure 8.**
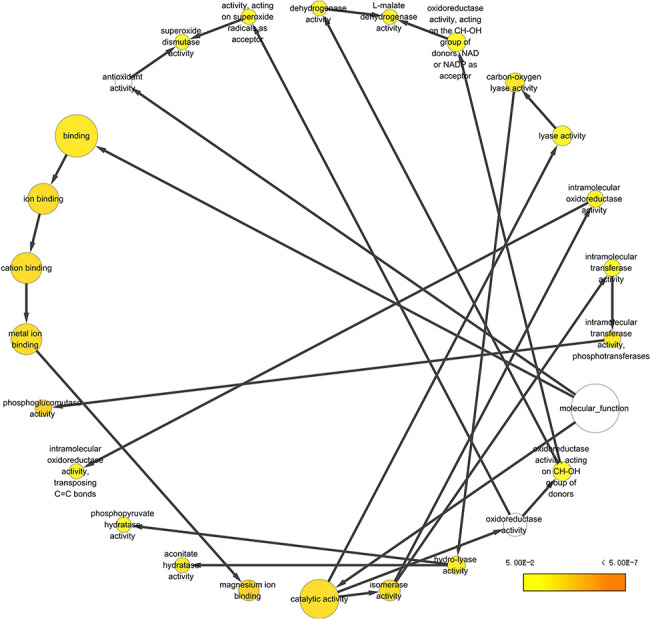
Example of GO annotation based on the 965 proteins common in five body fluids.

To take a closer look at this comparison, we focused on five body fluids that have the most protein counts, including blood, urine, CSF, SF) and BALF. An interesting discovery is that urine shares large numbers of common proteins with other fluids (Figure 7). A total of 4109, 3212, 2990 and 2950 proteins overlapped between the plasma and the other four body fluids (blood, CSF, SF and BALF, respectively). There are 965 proteins commonly detected in all five body fluids. The functional analysis using the BiNGO tool (183) in Cytoscape (184), reflecting information about cellular localization, molecular function and biological process of these proteins (Figure 8).

## Conclusions

The new HBFP database developed in this study represents the first of its kind as a comprehensive reference resource of HBFP. All data are available through an open-access user-friendly Web platform. All protein entries were manually curated, which can be easily traced back to the original literature. Users can query and download proteins of interest to verify discovery in their own study or conduct an *in silico* analysis on human secretomes. We currently schedule a regular update every 6 months. The future plan is to include computationally identified proteins using statistical and machine learning approaches (185–191). In the past decade, many computational studies have revealed unique strengths in overcoming challenges in profiling-based proteomics research in terms of discovering new protein bioavailability and functions. Those computationally predicted proteins can serve as a secondary resource for biomarker discovery. In summary, by providing a wealth of information and functional analysis, we believe the HBFP database can be an excellent tool for the research community to explore human proteome in various body fluids.
